# Steering Langevin Dynamics toward Transition States Using Collective-Variable-Free Resampling

**DOI:** 10.1021/acs.jctc.6c00679

**Published:** 2026-06-01

**Authors:** Michael Ketter, Georg K. H. Madsen

**Affiliations:** Institute of Materials Chemistry, TU Wien, A-1060 Vienna, Austria

## Abstract

Exploring the potential energy surface to sample transition-state regions is essential to understanding the atomic processes governing chemical reactivity. Ideally, the dividing surface between the educt and product states can be sampled without requiring predefined collective variables. Here, we adapt the stochastic saddle point dynamics (SSPD) algorithm by constraining the accessible configuration space according to the number of negative Hessian eigenvalues and evaluate its performance across increasingly complex systems. We motivate the adaptation using a simple two-dimensional model potential and demonstrate how the algorithm can efficiently sample the isomerization reaction of a Lennard-Jones cluster and the decomposition reactions of isopropyl alcohol. Combining the SSPD with automatically differentiable machine-learned interatomic potentials, we apply the approach to CO dissociation on a Co(001) surface both with and without explicit water solvation. The results highlight the role of SSPD as a framework for sampling transition-state regions in complex systems at finite temperatures and demonstrate its versatility in situations where it is not known a priori whether the reaction is governed by energetic or entropic contributions.

## Graphical abstract

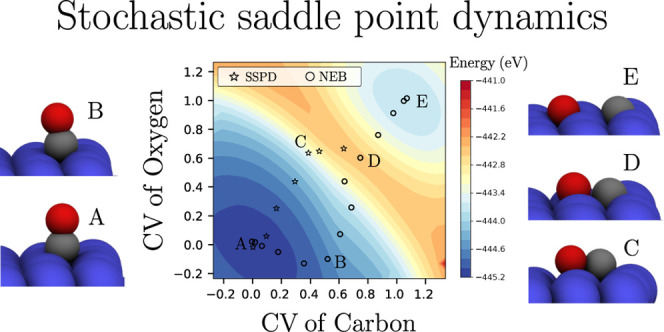

## Introduction

Chemical reactions are rare events where atomic configurations undergo a transition between metastable states on a potential energy surface (PES). Such transitions occur with low probability and are typically not sampled within the time scales of conventional molecular dynamics simulations. As a result, a wide range of approaches have been developed to characterize reaction mechanisms and estimate reaction rates without relying on brute-force dynamical sampling.

A common strategy is based on transition-state (TS) optimization methods combined with harmonic TS theory.^1^ In this framework, the reaction is described in terms of a minimum-energy path connecting predefined reactant and product states, and rates are determined from the properties of a first-order saddle point on the PES. While this approach has proven to be highly successful for simple systems, the underlying assumptions limit its applicability in complex chemical environments.

In particular, in the presence of an explicit solvent, reactive events are strongly coupled to collective environmental fluctuations, and the TS is more appropriately described as a thermally broadened region of configuration space rather than a single stationary point.^1^ Minimum-energy-path descriptions of liquid-phase reactions effectively impose a frozen environment,^2^ thereby suppressing solvent rearrangements and leading to an unrealistic description of solvent-mediated reactivity.

To address some of these limitations, TS-based approaches are often combined with free-energy-based enhanced sampling techniques, such as accelerated molecular dynamics^3^ or umbrella sampling.^4^ In such schemes, a predefined reaction pathway or collective variable (CV) is used to construct a low-dimensional representation of the reaction, along which the free-energy profile is computed. In complex systems, particularly in condensed phases, identifying a low-dimensional set of descriptors that captures the true reaction mechanism is nontrivial. Poor choices can obscure relevant pathways or introduce artificial barriers, and the requirement of prior knowledge limits the applicability of these methods for reaction discovery.

The stochastic saddle point dynamics (SSPD) algorithm, introduced by Lelievre and Parpas,^5^ provides an alternative framework that directly targets transition-state regions through stochastic temperature-dependent sampling. In contrast to conventional rare-event sampling techniques, SSPD does not require the definition of CVs. Instead, it explores the vicinity of a starting state (e.g., a local minimum) dynamically and uses the Hessian to assign weights to sampled configurations. Initially, weight-guided sampling drives the ensemble toward the TS region. Once the TS region is reached, frequent resampling prevents the ensemble from relaxing to the adjacent basins. While directly coupling the SSPD with ab initio methods such as density functional theory (DFT) is computationally prohibitive even for moderate system sizes, the emergence of machine-learned interatomic potentials (MLIPs) offers a promising route to overcoming this limitation. These models provide accurate and computationally efficient evaluation of energies as well as first- and higher-order derivatives through automatic differentiation,^6^ which enables the practical application of SSPD to complex systems.

In this work, we combine the SSPD with automatically differentiable MLIPs and investigate their applicability to the sampling of chemical reactions. To enable convergence toward reactive TSs, we modify the original algorithm by restricting the allowed configuration space according to the number of negative eigenvalues. With this adapted SSPD approach, we demonstrate that the reactive TSs of increasingly complex model systems can be sampled. We first introduce the SSPD algorithm, explain the idea and motivation behind the adaptation, and illustrate it by applying it to two simple systems: a seven-atom Lennard-Jones cluster and isopropanol, which features several rotamers separated by low-lying saddle points. We then extend the analysis to CO dissociation on Co(001) with and without explicit water solvation and emphasize the SSPD’s role as a framework for sampling transition-state regions in complex systems under finite temperature conditions.

## Stochastic Saddle Point Dynamics

### Algorithm

The search for index-1 saddle points is generally formulated as a min–max optimization problem, with maximization along the unstable direction and minimization along all remaining dimensions. In the SSPD algorithm, this problem is addressed by evolving an ensemble of configurations through stochastic minimization coupled to a resampling scheme that favors regions likely to contain saddle points.^5^ The algorithm achieves this by solving a set of coupled stochastic differential equations

1 $$dX_{t}^{\left(i\right)}=-\nabla V\left(X_{t}^{\left(i\right)}\right)dt+\sqrt{2\beta^{-1}}dB_{t}$$

2 $$dY_{t}^{\left(i\right)}=-\nabla^{2}V\left(X_{t}^{\left(i\right)}\right)Y_{t}^{\left(i\right)}dt$$

which represent a stochastic approximation of the time evolution of the probability current between two metastable PES minima.^7,8^ In eq 1, ${\left(B_{t}\right)}_{t\geq 0}$ denotes a *d*-dimensional Brownian motion, which introduces stochastic fluctuations corresponding to thermal noise at the inverse temperature β. The underlying distribution is represented by an ensemble ${\left\{X^{\left(i\right)},Y^{\left(i\right)}\right\}}_{i=1}^{N}$ , where *X* ^(*i*)^ labels instances of the configuration space ensemble and *Y* ^(*i*)^ labels instances of the weight space ensemble.

In the low temperature limit, the current has its maximum at an index-1 saddle point.^5,9^ At higher temperatures, ref 10 has previously defined the surface of maximal probability current and investigated its theoretical properties. The SSPD algorithm can thus be viewed as implementing the min–max optimization. Sampling the configuration space according to eq 1 corresponds to performing overdamped Langevin dynamics and can be interpreted as stochastic minimization of the PES. At the same time, the resampling step based on ∥*Y* ^(*i*)^∥ effectively performs a maximization toward sampling configuration space with a high probability current. Taken together, these two components drive the system toward convergence to a transition-state region.

In principle, the configuration space could be resampled according to ∥*Y* ^(*i*)^∥ in every iteration. However, to ensure that resampling is only performed when necessary, ref 5 suggests using the effective sample size (ESS) as a proxy for the resampling necessity. The ESS of the ensemble distribution ∥*Y* ^(*i*)^∥ is approximated by

3 $$ESS\left(\omega^{\left(1\right)},\omega^{\left(2\right)},...,\omega^{\left(N\right)}\right)=\frac{1}{\sum_{i=1}^{N}{\left(\omega^{\left(i\right)}\right)}^{2}}$$

where the weight ω^(*i*)^ denotes the normalized version of ∥*Y* ^(*i*)^∥. If the ESS falls below a certain threshold, a resampling step is performed. A natural choice for the resampling algorithm is stratified resampling, where the probability for resampling each configuration is proportional to its weight. It ensures that the configuration space ensemble is steadily shifted to regions with high weight. Additionally, stratified resampling is known to be robust against deterioration of the ensemble diversity, which is especially helpful in the high-dimensional case of atomistic simulations. In practice, we observed two competing effects associated with the choice of the ESS threshold. For high thresholds, resampling was triggered too early, limiting the exploration of the configuration space and preventing convergence toward reactive TS regions. For low thresholds, resampling occurred too infrequently to maintain the ensemble near the TS region without sliding into one of the adjacent minima. As a compromise, we found that resampling when the ESS, eq 3, fell below 0.99*N*, where *N* denotes the ensemble size, provides a robust balance between exploration and confinement (see Supporting Information for further details).

### Implementation

The present implementation of the SSPD is strongly influenced by the one provided in ref 5. Just-in-time compilation, as implemented in JAX,^11^ was used for optimizing the performance of the algorithm. All *Y* ^(*i*)^ were initialized by randomly sampling a vector of length 3*n*_at_ from the uniform distribution of range 0 to 1. After that, each vector was normalized by dividing by its 2-norm. For running the dynamics, eqs 1 and 2 were discretized in time using an explicit Euler–Maruyama scheme.^5^ First- and second-order derivatives of the potential were evaluated using automatic differentiation. After updating all *X* ^(*i*)^ and *Y* ^(*i*)^, the corresponding weights were calculated by taking the 2-norm of the *Y* ^(*i*)^. In order to prevent under- or overflowing, the weights ω^(*i*)^ were normalized employing the log-sum-exp trick. All simulations were conducted with an ensemble size of 200 configurations. The inverse temperature β was individually fixed for each system.

The acceptance rate of the configuration space restriction is strongly interconnected with the length of the time step used for the overdamped Langevin dynamics. After defining the initial time step specifically for each test system, it was adjusted dynamically in each iteration of the SSPD. For this, a range of allowed acceptance rates was predefined for each investigated system. If the acceptance rate in a given overdamped Langevin dynamics iteration fell below the allowed range, the time step for the subsequent overdamped Langevin steps was decreased by a factor of 0.99. In the case of overshooting the allowed acceptance range, the time step was increased by the factor 1.01.

### Configuration Space Restriction

The implementation based on ref 5 did not yield the expected convergence to reactive saddle points, particularly in systems with multiple low-energy nonreactive saddle points. We attribute the failure to the following observation. Assuming a fixed configuration *X* ^(*i*)^, the Hessian constitutes a 3*n*_at_ × 3*n*_at_ matrix. The dynamic process in weight space then corresponds to a homogeneous system of differential equations. This allows one to formulate an analytical solution for eq 2 as

4 $$Y_{t}^{\left(i\right)}=\sum_{j=1}^{3n_{at}}v_{j}^{\left(i\right)}e^{-\lambda_{j}^{\left(i\right)}t}$$

where *v*_*j*_ denotes the eigenvectors of the Hessian and λ_*j*_ the corresponding eigenvalues. It can be observed that the exponential part acts as an amplification or damping factor of the eigenvector *v*_*j*_, depending on the sign of the corresponding eigenvalue λ_*j*_ of the Hessian matrix. Thus, the norm of the solution ∥*Y*_*t*_^(*i*)^∥ gets amplified exponentially in regions with one or more negative eigenvalues. Additionally, more eigenvectors are amplified the higher the number of negative eigenvalues of the Hessian is. In practice, this led to frequent resampling in regions with several negative eigenvalues close to the nonreactive saddle points. To mitigate this issue, the explored configuration space was restricted to regions where the Hessian has at most one negative eigenvalue by rejecting all steps leaving the allowed region.

To illustrate the influence of restricting the configuration space, we apply the algorithm to the 2D toy potential shown in Figure 1. The potential resembles a 2D double-well potential with a “bump” near one of the minima. The two minima are separated by an index-1 TS region, while the bump induces a circular region with two negative eigenvalues of the Hessian near the left minimum. Initializing the SSPD algorithm in the left basin, 5000 iterations with and without the configuration space restriction were performed for a fixed β^–1^ = 15.0. Without the restriction, the ensemble gets stuck around the region with two negative eigenvalues (Figure 1, left). By contrast, enforcing the restriction means that the ensemble moves toward the saddle point region between the two potential energy minima (Figure 1, right). This shows that the restriction is effective in situations where regions with multiple negative Hessian eigenvalues are present.

**Figure 1:**
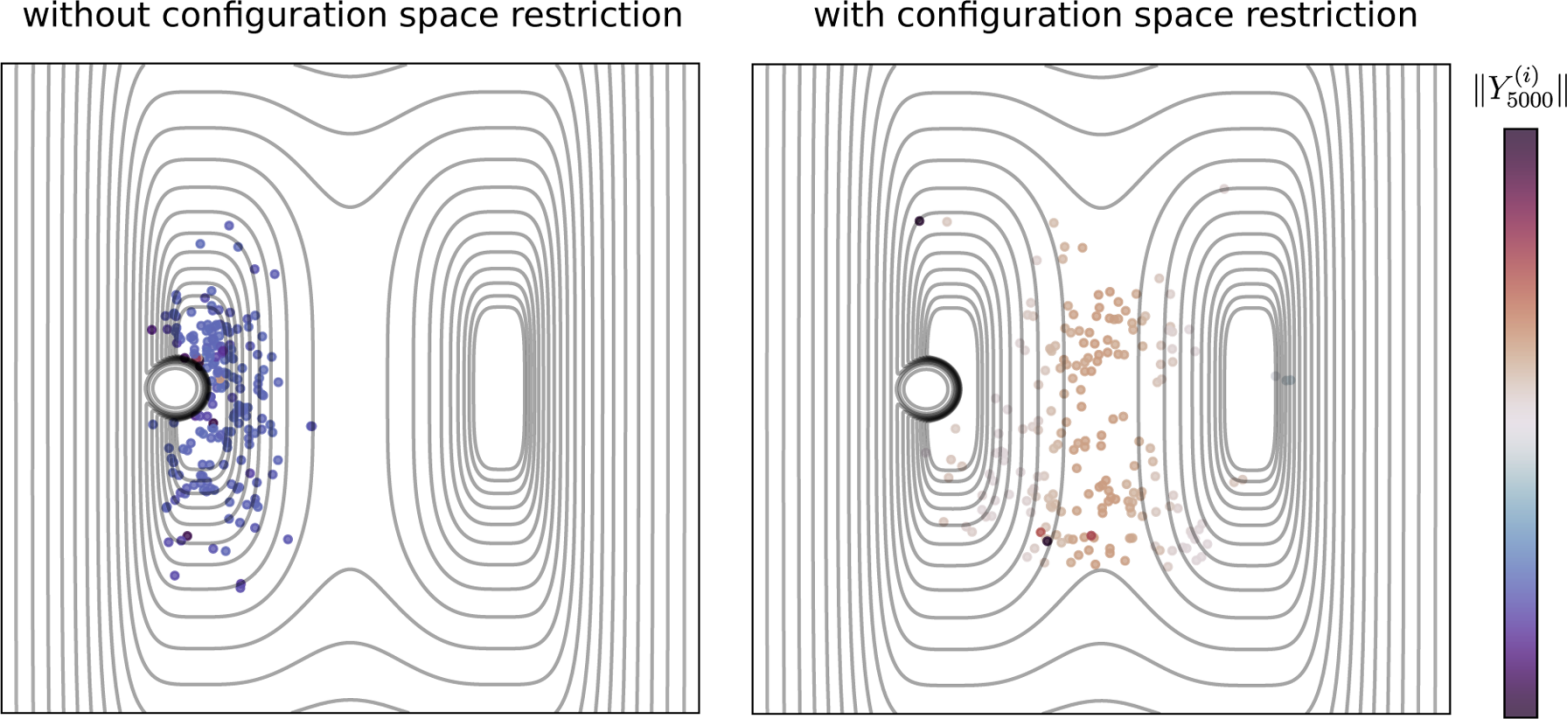
2D toy potential, $V\left(x,y\right)={\left(x-3\right)}^{4}+y^{4}+200\;\exp\left(-{\left(10{\left(x+2\right)}^{2}+10y^{2}\right)}^{4}\right))$ and the distribution after 5000 iterations without restricting the configuration space (left) and with restricting the configuration space (right). An illustration depicting the number of negative eigenvalues of different configuration space regions is available in theSupporting Information.

## Computational Details

### MLIP Training

MACE version 0.3.12 ^12,13^ models were employed as surrogates for energy, force, and Hessian^6^ evaluations. The training of the models was performed using the provided package. Deviations of the default hyperparameter set are described in the following. For descriptor generation, a cutoff radius of 5.0 Å was applied. For message passing, 64 invariant messages and 64 equivariant messages were used. Minimization in the training process was performed using the AMSGrad optimizer with default parameters. An exponential moving average was used with a decay of 0.99. A loss scheduler similar to that described in ref 14 was utilized. The optimization was run for a maximum of 1500 epochs with an early stopping patience of 250, a force weight of 100.0, and an energy weight of 1.0. After that, another 500 epochs were performed with a force weight of 100.0 and an energy weight of 1000.0. It should be noted that while needing higher-order derivatives for the stochastic saddle point dynamics, only energies and forces were fitted.

### Isopropanol Training Data

Five different isopropanol decomposition reactions were extracted from the Transition-1x data set,^15^ each consisting of eight intermediate NEB images between the educt and product states. To capture the configuration space surrounding the minimum energy path, each of the 40 configurations was randomly perturbed. In order to prevent artifacts due to artificial minima in the MLIP at large bond distances, additional configurations were generated by stepwise elongation of the relevant bonds. A small perturbation was added to all positions of those structures as well. Combining the configurations, this resulted in training and validation data sets containing 3556 and 1200 configurations, respectively. The energies and forces were calculated using ORCA 5.0.2 ^16^ applying the ωB97x functional^17^ and 6-31G­(d) basis set.^18^ This is consistent with the level of theory used in the original Transition-1x data set.

### Training Data for CO Dissociation on Co(001)

Educt, product, and TS structures for CO dissociation adsorbed on an fcc- and an hcp-site of Co(001) were obtained from ref 19. By randomly perturbing those six structures, a first data set and subsequently an initial MLIP were generated. Using this MLIP, further configurations were sampled via NEB search along the paths of the fcc- and hcp-dissociation reactions as well as from the CO migration path connecting an fcc- with an hcp-site. To prevent artificial expulsion of the oxygen atom from the CO-molecule, structures were generated by stepwise elongation of the C–O bond of a molecule adsorbed at an hcp- as well as an fcc-site. All of these configurations were randomly perturbed, added to the initial training set, and used to train the final model. The final training and validation data sets contained 297 and 97 configurations, respectively. The energies and forces of the configurations were calculated using VASP version 6.4.2 ^20^ with the PBE functional^21^ and an energy cutoff of 400 eV. All other DFT settings were chosen equivalent to the ones used in ref 19.

### Training Data for CO Dissociation on Co(001) in an Explicit Water Solvent

In order to make use of existing databases for water, we added 1588 pure water structures initially published by ref 22 to the database used for modeling the unsolvated system. Those structures have been shown to provide a good starting point for modeling solvated interfaces.^23^ To include some information about the solvated interface, we added 30 and 24 structures, respectively, generated via two different approaches: (1) based on the Co-slab with CO adsorbed at an fcc-site, 43 randomly rotated water molecules were added at random positions with the restriction that their nearest neighbor should be at least 2 Å apart. (2) Based on randomly sampled structures from one CO-on-Co SSPD run without solvation, 43 water molecules were added randomly using the same restriction as above. After adding the water molecules, a slight random perturbation was added to all positions. The final training and validation data sets contained 1957 and 519 configurations, respectively. Due to the added solvation, we recomputed the energies and forces of the full database using VASP version 6.4.2 ^20^ with the RPBE functional and an energy cutoff of 850 eV. Additionally, we applied the D3 correction^24^ with a zero damping scheme to all atoms and used hard projector augmented wave pseudopotentials^25^ for hydrogen, oxygen, and carbon. All other DFT settings were chosen equivalent to the ones used in ref 26.

### Simulation Settings for SSPD

In the 3D Lennard-Jones cluster example, we initialized the SSPD in the lowest state and performed 5000 iterations with an initial overdamped Langevin dynamics time step of 0.005 and a fixed β^–1^ = 0.15. For the configuration space restriction, the minimal and maximal acceptance ratios were set to 0.35 and 0.55, respectively.

In the isopropanol examples, we performed a total of 50 SSPD runs with 5000 iterations each, an initial time step of 0.0075 fs, and a fixed acceptance rate interval between 0.5 and 0.8. The low time step compared to regular overdamped Langevin dynamic simulations was chosen because TS configurations decay rapidly. Using a small time step enables the algorithm to detect a TS and shift the ensemble into the corresponding configuration space region before it decays. To observe the sought transitions in the applicable simulation time, the system temperature was increased. Five different temperatures in the range from 7542 to 9863 K (β^–1^ = 0.65–0.85 eV) were used, each with ten different initial random seeds, resulting in 50 independent SSPD runs.

For the CO-dissociation on Co(001), we initialized the SSPD runs with a 4 × 4 Co slab where CO was adsorbed at one of the fcc-sites. The SSPD ensemble was then propagated for 5000 iterations with an initial time step of 0.01 fs and a fixed β^–1^ = 0.65 eV. The minimum and maximum acceptance ratios for the configuration-space restriction were set to 0.5 and 1.0, respectively. The system size of 66 atoms, corresponding to 198 degrees of freedom, made it inefficient to calculate the full Hessian in each SSPD iteration. To improve the efficiency, we used an approach where only a 6 × 6 submatrix of the full Hessian, restricted to the degrees of freedom of the CO molecule, was employed for updating the sampling weights. Additional analysis validating this approximation is provided in the Supporting Information.

To initialize the solvated CO-dissociation on Co(001) simulations, we reused the initial configuration of the unsolvated system and added 43 water molecules in the same fashion as for the generation of the MLIP training structures. To ensure identical DFT settings, simulations for both the unsolvated and the solvated system were performed using the MLIP generated with the solvated CO-dissociation-on-Co(001) training data. We restricted the reactive subdomain to the CO-molecule, used an initial time step of 0.05 fs, and fixed *T*_react_ = 7542 K. The minimal and maximal acceptance ratios for the configuration space restriction were set to 0.5 and 1.0, respectively. For both chemical systems, we performed 1000 SSPD iterations before we annealed *T*_react_ → *T*_sample_ = 450 K in the course of 1000 subsequent iterations. By keeping the resampling enabled for 5000 additional iterations, we allowed the surroundings of the CO molecule some time to respond to the reactive perturbation. Deactivating the resampling mechanism, we performed 6000 iterations of downhill sampling with a fixed time step of 0.05 fs.

## Results and Discussion

### 3D Lennard-Jones Cluster

In order to show that the algorithm works as intended in high-dimensional systems, we use the 3D Lennard-Jones cluster with seven atoms (LJ-7)^27^ as the first test system. For the Lennard-Jones potential, the parameters were set as ϵ = 1.0 and σ = 2^–1/6^. As can be seen in the NEB search between the two lowest energy conformers visualized in Figure 2 (right), they are connected by a potential energy barrier of 1.06.

**Figure 2:**
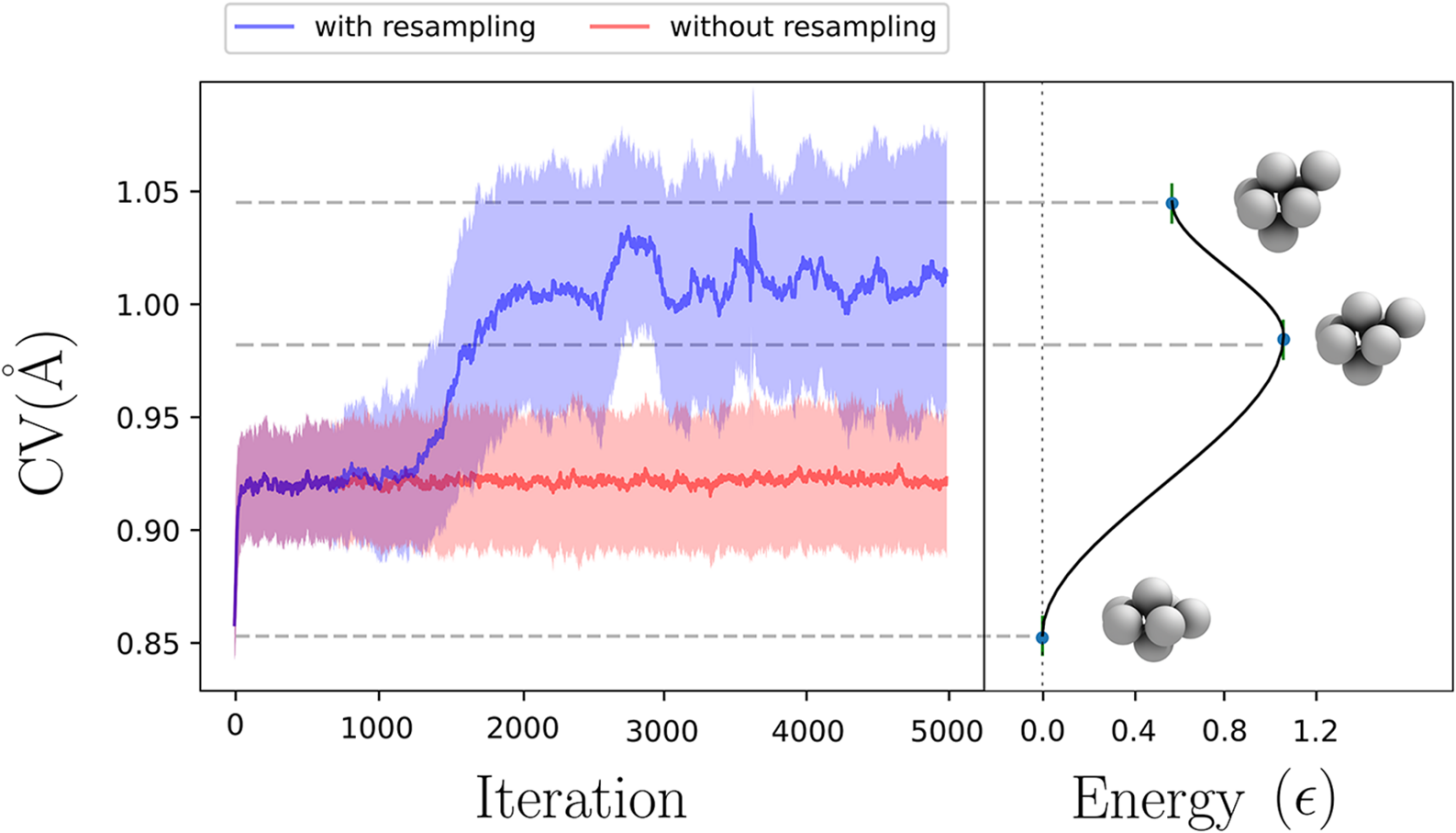
Left: Evolution of the trend of the CV during the SSPD of the LJ-7 cluster (blue). The distance between the center of mass and the outermost atom is used as a CV. For comparison, a trajectory with resampling switched off is shown (red). Solid lines depict the ensemble-averaged CV, while the shaded regions indicate the corresponding standard deviation colored in light. Right: Illustration of an NEB search connecting the two relevant minima of the LJ-7 cluster. Visualizations of the educt, product, and TS configurations are shown.

To illustrate how the resampling steps drive the ensemble toward the TS region, two simulations were performed where resampling was switched on and off, respectively. While the former case corresponds to solving the coupled equations, eqs 1 and 2, the latter case corresponds to propagating the system according to Langevin dynamics governed solely by eq 1. Both simulations were initialized by using the same random seed. Using the distance between the center of mass and the outermost atom as a CV for the transition, the behavior of both runs is illustrated in Figure 2 (left). For comparison, the CVs of the educt, product, and TS structure obtained through a NEB search are indicated as well. It should be noted that the CV is only introduced for illustrative purposes and does not have any influence on the dynamics during the simulation. The ensemble, where resampling was switched off, reached a steady state far from the TS region after a short initial period. When resampling was enabled, the ensemble followed the same trend for the first 1000 iterations. After that, the distribution started to drift to higher CV values, and its mean converged to a value between the TS and the product. It has to be noted that due to temperature effects, convergence to a slightly higher CV value compared to the NEB search is expected.

To better understand how the algorithm shifts the ensemble toward the TS region, conjugate gradient energy minimizations were used to associate each configuration in the ensemble to one of the two minima. Figure 3 (top) depicts the fraction of configurations that can be associated with either the educt (green) or the product (blue) minimum as a function of the performed iterations. To correlate those trends with the resampling process, the standard deviation of the resampling weights as a function of the performed iteration is shown in the middle panel. Additionally, the ensemble mean and spread of the smallest Hessian eigenvalue are shown in the bottom panel.

**Figure 3:**
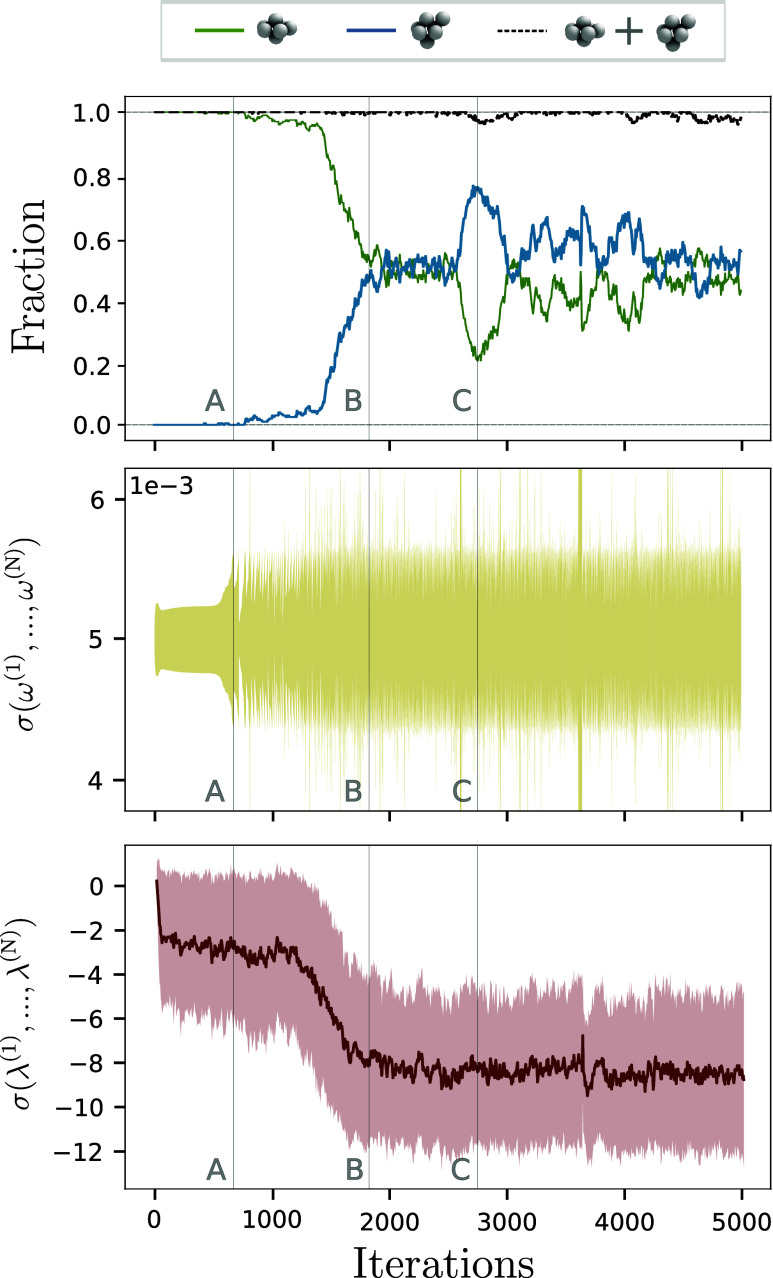
Top: Fraction of the ensemble assigned, by conjugate-gradient minimization, with the educt minimum (green), the product minimum (blue), and the sum of both (black). Middle: Time evolution of the resampling weights spread. Bottom: Time evolution of the mean and standard deviation of the lowest Hessian eigenvalues for the ensemble members. The points A, B, and C correspond to events discussed in the text.

As can be seen in the middle panel, the spread of the resampling weights remains constant for ∼500 iterations. After that, the resampling weight distribution starts to spread out until a threshold is passed. This triggers a resampling step (A), which tightens the resampling weight distribution again. Comparing this with the top panel, it can be seen that shortly prior to the first resampling step, configurations associated with the product minimum emerge. From this point on, resampling is performed very frequently, reducing the ensemble mean of the lowest eigenvalue more and more. The top panel shows that after 2000 iterations (B), approximately half of the distribution can be associated with the educt and the other half with the product minimum. This coincides with Figure 2, where the mean CV reaches the TS value after 2000 iterations as well.

At later iteration counts, the ensemble composition becomes unbalanced at one point (C). This correlates with the observation that not all configurations in the distribution can be associated with either the educt or product minimum. Since several TSs connect the product minimum with other metastable minima,^28^ it is most likely that the algorithm has detected one of the consecutive saddle points and tries to resample the ensemble accordingly. However, after a while, the algorithm steers the ensemble back to a region where half of its members can be associated with the educt and the product minimum, respectively.

### Isopropanol Decomposition

To show that the SSPD can be combined with MLIPs for finding TSs, we investigated isopropanol. Realistic compounds add further difficulties to the problem of identifying reactive TSs. In the case of isopropanol, the vicinity of the ground state contains several rotamers, separated by low-lying saddle points. The algorithm must therefore be controllable to skip these nonreactive saddle points and instead target higher lying reactive ones. Within the SSPD algorithm, this control is achieved by adjusting the resampling threshold, which determines how sensitive the algorithm is to changes in resampling weights. For isopropanol, several threshold values were tested, and a value of 0.99*N* was found to effectively suppress convergence to rotameric saddle points while still allowing the discovery of reactive TSs.

The TSs found with the corresponding MLIP barrier heights are depicted in Figure 4. An additional analysis looking at the distribution of the final SSPD ensembles can be found in the Supporting Information. The reactions corresponding to TS1, TS3, and TS4 are the three TS below 5 eV included in the Transition1x data set. In addition, eight of the 40 runs with β^–1^ > 0.65 eV converged to the TS2 reaction, which is a rearrangement to propanol. Since TS2 is not part of the Transition1x database, it was validated using a DFT-backed NEB search. Thereby, the TS was confirmed exhibiting a DFT barrier height of 5.12 eV. The rediscovery of the three isopropanol decomposition reactions with the lowest barrier heights in the Transition1x data set, in addition to the discovery of a new TS not present in the MLIPs training data set, demonstrates the SSPD’s ability to find previously unknown TSs.

**Figure 4:**
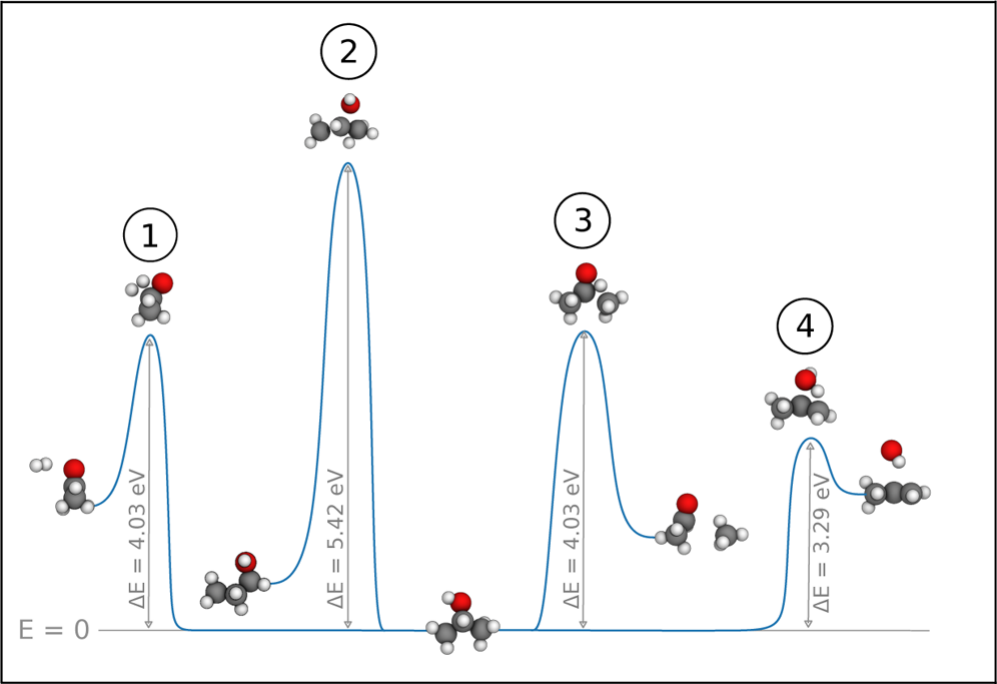
Four found reactions with the associated MLIP barrier heights.

### CO Dissociation on Co(001)

Next, we demonstrate that the algorithm can be applied to study complex surface reactions in catalytically active materials. Following ref 19, the test system was the CO dissociation reaction on a Co(001) slab. A bulk-terminated 4 × 4 surface with four Co layers and NEB searches was used to study a mechanism, where CO is initially adsorbed at one of the fcc-sites and subsequently cleaves. In the resulting product configuration, the carbon and oxygen atoms occupy an hcp- and fcc-site, respectively.

To illustrate how resampling drives the ensemble toward the TS region, the CO distance was used as a CV. Figure 5a compares the CV evolution for simulation runs with enabled and disabled resampling. Without resampling, the ensemble remains confined to the initial minimum throughout the simulation. When resampling is enabled, the CV behavior indicates that the ensemble converges to the TS region after approximately 2000 iterations.

**Figure 5:**
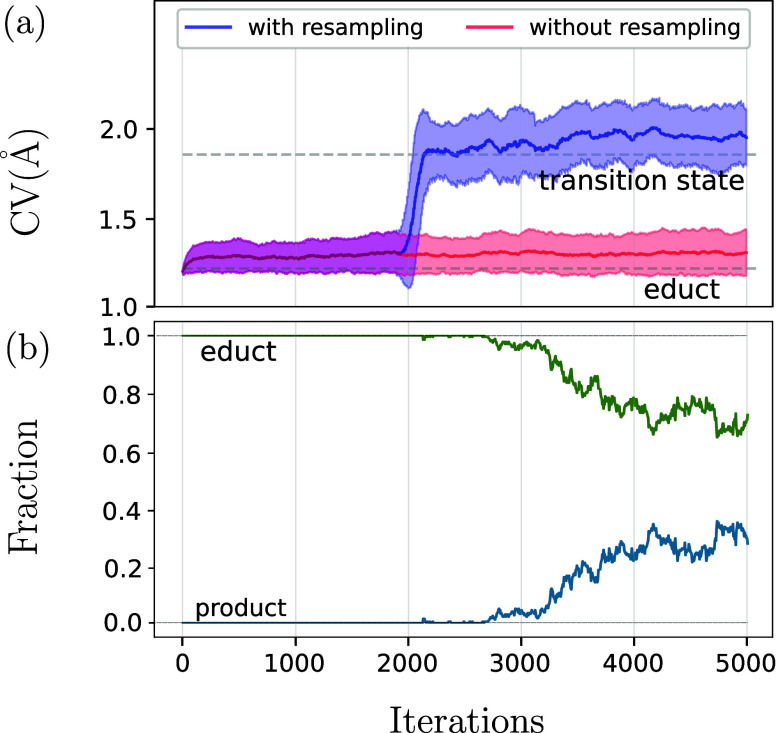
(a) Illustration of the trend of the C–O distance CV for stochastic saddle point dynamics (blue) and overdamped Langevin dynamics (red). CV values of the educt and TS were taken from ref 19. (b) Fraction of the ensemble assigned, by conjugate-gradient minimization, with the educt minimum (green), the product minimum (blue), and the sum of both (black).

To further analyze convergence, conjugate-gradient energy minimizations were used to determine the fraction of the ensemble associated with the educt and product minimum. Figure 5b depicts the evolution of both fractions along the trajectory. Interestingly, at iteration 2000, almost no ensemble member can be associated with the product, contradicting the impression obtained from the CV. Only after about 3000 iterations does the proportion of ensemble members reaching the product minimum increase significantly.

To understand this discrepancy, we first perform a NEB search connecting the educt and product minima (structures A and E in Figure 6). The resulting minimum-energy path from the educt minimum to the TS is composed of two consecutive CO movements. In the first step, the CO molecule migrates from an fcc site toward a neighboring hcp site (structure C in Figure 6). In a second step, the CO molecule tilts toward the surface, and the C–O bond starts to break (structure D in Figure 6).

**Figure 6:**
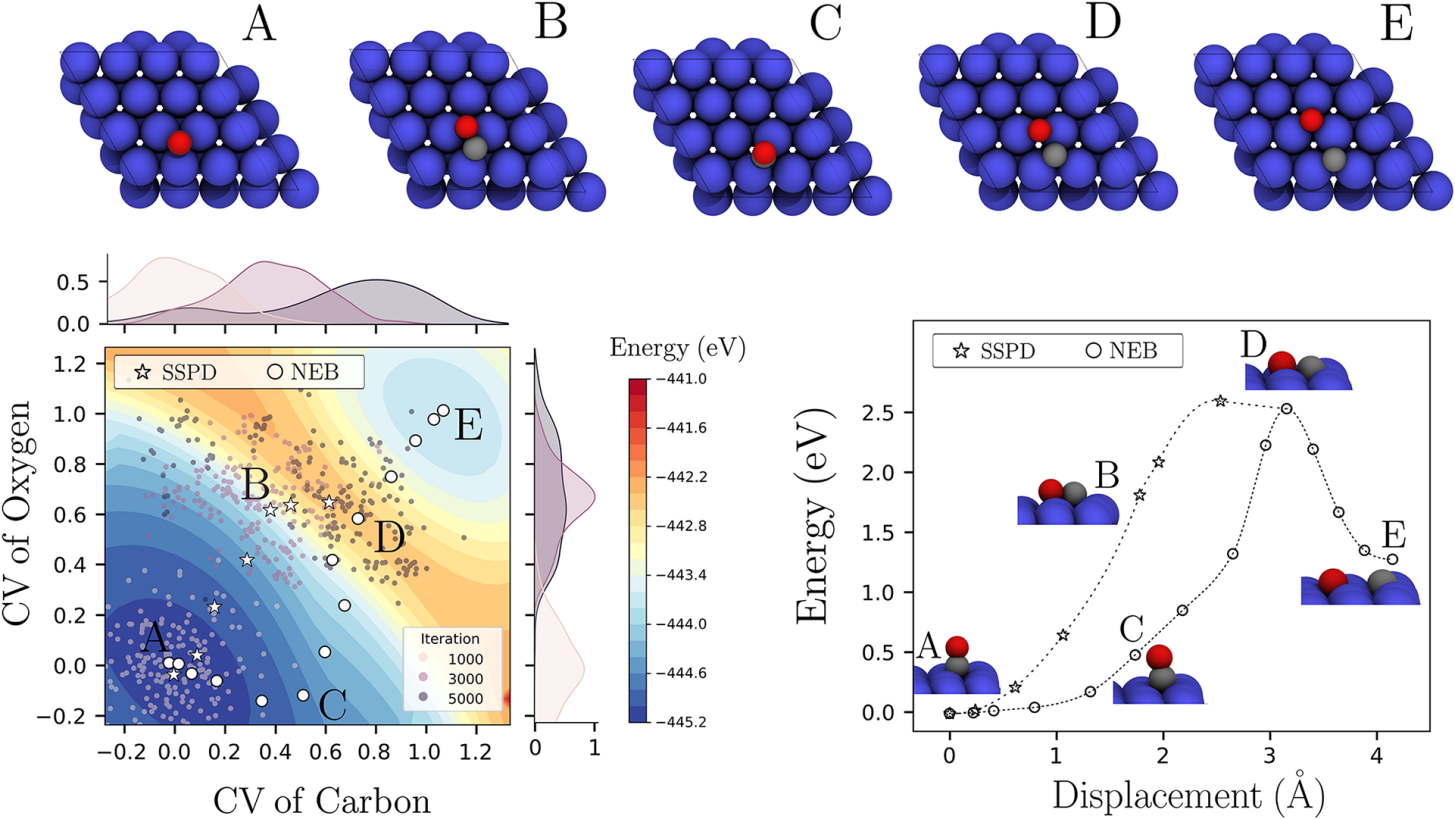
In the upper row, five different configurations (A–E), corresponding to distinct regions on the potential energy surface, are depicted. The left plot in the lower row visualizes the 2D potential energy surface of the CO dissociation reaction and compares the NEB (open circles) with the SSPD (stars) path. Therein, scatter plots and density curves illustrate the SSPD ensemble at three iteration counts. The right plot in the lower row depicts the energy profiles of the NEB and SSPD trajectories and illustrates changes of the configurations along the paths.

In light of the two consecutive movements, we characterize the reaction in terms of two CVs. The first CV is defined as a linear interpolation between the educt and product carbon *x* – *y* positions, while the second CV is defined analogously for the oxygen atom. By constraining these CVs to fixed values and relaxing the remaining degrees of freedom, a 2D PES of the reaction was obtained and is depicted in the left part of Figure 6. As expected, the NEB path on the 2D PES connects the educt minimum (A) to the product minimum (E) via the TS (D). Starting from the educt configuration (A), the NEB trajectory takes a very gentle ascent via the intermediate CO-migration structure (C) before reaching the TS (D).

The left panel of Figure 6 also shows scatter plots of the SSPD ensembles for iterations 1000, 3000, and 5000 (see also Figure 5). The projections of the ensembles onto the two CVs are shown on the axes. While the overall spread of the ensembles is substantial, the mean of the distributions (indicated by stars) clearly converges to the TS. In order to concentrate the ensemble around the TS region, an annealing schedule can be applied following the first resampling step; a representative protocol and its effects are discussed in the Supporting Information.

Notably, the SSPD trajectory converges toward the TS following a path that is reversed compared to the NEB result. At first, the CO molecule tilts toward the surface, accompanied by a C–O elongation (structure B in Figure 6). Subsequently, the carbon atom migrates to an hcp site (structure D in Figure 6). The reversed order of movement is consistent with the observation that the average CO bond length increases after approximately 2000 iterations (Figure 5a) while nearly all ensemble members are still associated with the educt minimum. This suggests the tilting and bond elongation drives the ensemble into a region of the PES with negative eigenvalues of the Hessian, thereby triggering the resampling process. The early onset of the CO tilting is reflected in the right panel of Figure 6, where the energy profiles of the NEB and SSPD pathways are compared. Subsequent weighted diffusion (eqs 1 and 2) migrates the carbon to the hcp site, ultimately resulting in convergence to the same TS as identified with NEB.

In general, the differences between the NEB and SSPD paths can be interpreted as an influence of finite temperature effects. Due to the static approach, NEB finds the minimum energy path associated with a reaction occurring at 0 K. On the other hand, SSPD operates at finite (usually elevated) temperatures, thereby favoring paths that reflect not only energetic but also entropic contributions.

### CO Dissociation on Co(001) in an Explicit Water Solvent

To test the SSPD in situations where entropic effects play a more pronounced role, we extend the CO dissociation on Co(001) example by including explicit water solvation. We then compare the behavior with and without added water solvation using a two-stage simulation scheme consisting of an activation and a sampling phase. In the activation phase, the ensemble is initialized in the vicinity of a potential-energy minimum. The reactive subsystem (e.g., the CO molecule) is driven toward the transition region by increasing its effective temperature, while the rest of the system evolves at the lower target temperature. Using the SSPD weights to guide resampling, the ensemble converges toward the TS region. Upon convergence, the reactive subsystem is steadily annealed until its temperature matches that of its surroundings.

We performed simulations for both the solvated and unsolvated systems, with the target temperature set to *T* = 450 K. Reusing the CO distance as the CV, the evolution of its distribution after reaching the temperature of interest and stopping the resampling is plotted in steps of 1000 iterations in Figure 7b,c for both systems. Both systems initially yield a CV distribution centered around 2.0 Å, consistent with the transition-state region of the previous example in Figure 5. As the systems relax, both adjacent potential energy minima at CO distances below 1.5 Å and above 2.5 Å become increasingly populated. The relaxation toward the intact adsorbed structure occurs rapidly, whereas the progression toward CO dissociation is significantly slower. Interestingly, when comparing the last iteration distributions (Figure 7b,c in dark), the solvation seems to stabilize the split CO molecule at shorter distances.

**Figure 7:**
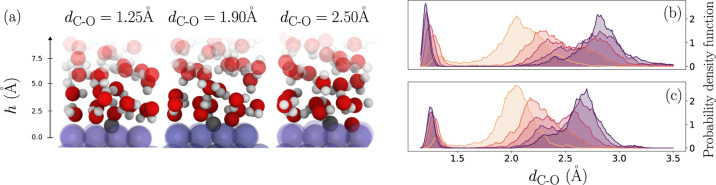
(a) Selected configurations from the educt, transition state, and product regions of the CO-dissociation reaction with the explicit water solvent. (b,c) Histograms depicting the change of the ensembles’ C–O distance in steps of 1000 iterations (from light to dark) during the relaxation. The two plots correspond to the systems: (b) CO on Co(001) and (c) CO on Co(001) with explicit water solvation.

Using the configurations from the sampling phase, we investigate the response of the water solvation layer to the progressing reaction. For that, Figure 8 depicts the oxygen density of the water solvation layer as a function of the CO distance and the distance *h* to the topmost Co atoms in the slab. Selected configurations at both reaction ends and the TS region are depicted in Figure 7a. The interfacial water structure exhibits four distinct density features, roughly 2.25 Å, 3.25 Å, 4.75 Å, and 7.1 Å away from the surface. While the features at 2.25 Å, 3.25 Å, and 7.1 Å are characteristic of water at flat surfaces,^29^ the feature at 4.75 Å stems from the perturbation of the solvent by the adsorbed CO molecule. The presence of the CO molecule pushes surrounding water molecules away from the surface slab, as can also be seen in the configuration depicted in Figure 7a. It should be noted that data is available only sparsely for distances around 1.5 Å due to the fast decay of those states. When analyzing the density differences along the CO distances, one finds that the feature corresponding to the perturbation of the solvation shell merges with the 3.25 Å feature at CO distances larger than 2.0 Å. Intuitively, this can be explained by a decreased interaction between the CO molecule and the solvation shell due to the increasing tilt of the molecule toward the surface during the reaction. Interestingly, the two affected density features are still completely disconnected at the transition state region $\left(d_{C-O}\approx 2.0\;Å\right)$ even though the CO molecule lies almost completely flat, see Figure 7a middle structure. This suggests that the relaxation of the perturbed solvation shell happens as a subsequent step after the transition region is left.

**Figure 8:**
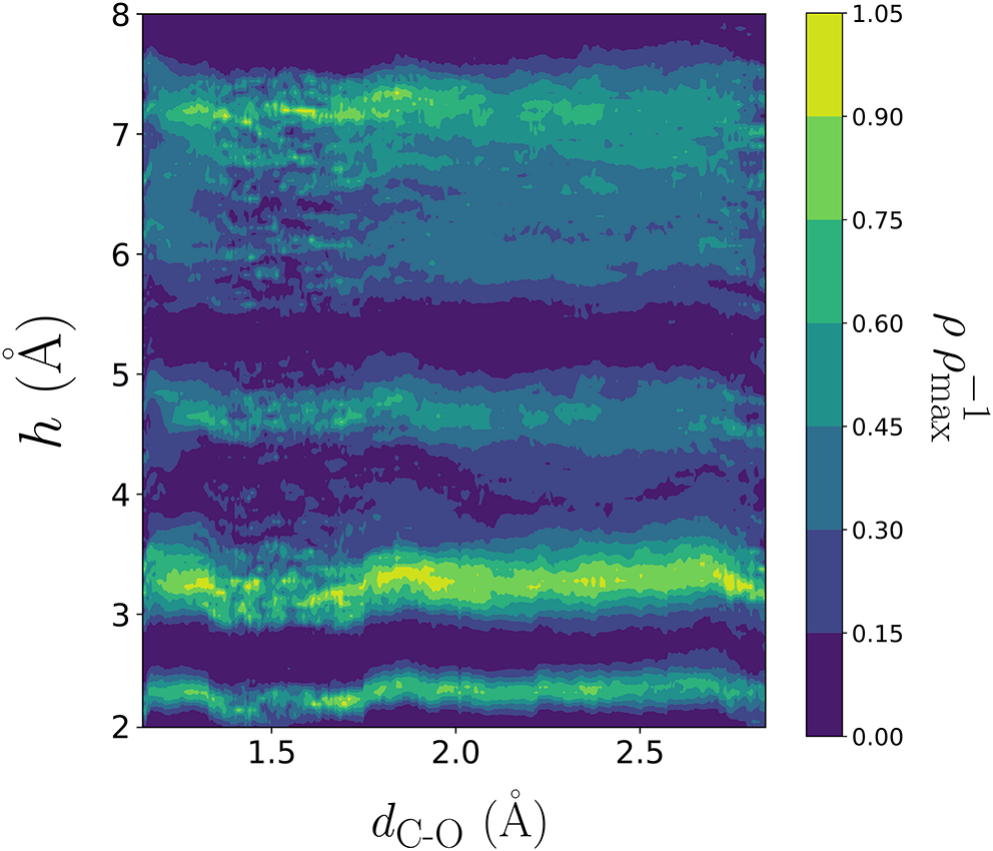
Oxygen density as a function of the CO distance and the distance from the slab surface in the *z*-direction.

In this context, SSPD can also be viewed in the spirit of downhill sampling techniques for elucidating reaction mechanisms in condensed systems.^30^ In such approaches, mechanistic information about the reaction is gathered by propagating trajectories downhill from a suitably defined transition state region. Conventionally, initial configurations are generated by solvating gas-phase transition states using appropriate schemes^31^ and assigning momenta using quasi-classical sampling.^32^ In contrast, SSPD enables direct identification of TS regions in the presence of explicit solvation, providing a consistent framework for studying solvent-coupled reactivity.

## Conclusion and Summary

We combined an adapted version of SSPD with automatically differentiable MLIPs and successfully tested them in various chemical settings. Starting from a configuration located in an arbitrary potential energy minimum, the algorithm enables an exploratory search for unknown adjacent transition state regions. By construction, SSPD samples the configuration space region with the highest probability flux in the quasi-equilibrium situation, making it a versatile tool for reaction discovery in complex chemical systems.^5,7,8^ We have illustrated how SSPD, in the spirit of downhill sampling techniques, offers a robust framework for elucidating the reaction mechanism in condensed systems by directly identifying TS regions under explicit solvation, thereby enabling a consistent description of solvent-coupled reactivity.

The SSPD presents an enhanced sampling technique which does not rely on incorporating prior chemical domain knowledge into a CV. Emerging foundation MLIPs^33–35^ have demonstrated remarkable success across a wide range of materials science problems. However, most of the data available in public databases for training corresponds to equilibrium situations. As a result, such models perform well in regions around potential energy minima^36^ but can struggle to accurately capture reactive properties. Due to its exploratory nature, the SSPD is inherently compatible with active-learning approaches and we anticipate that MLIPs can be systematically improved by sampling directly from SSPD trajectories.

## Supplementary Material



## Data Availability

The code used to run the SSPD dynamics can be found and cloned from https://github.com/Madsen-s-research-group/stochastic-saddle-point-dynamics. The training data set, models, initial structures, generated SSPD trajectories, and a script showing how the 2D potential energy surface was generated in the CO-dissociation example using the two obtained CVs are available on Zenodo at 10.5281/zenodo.17434312.

## Notes

Additional analysis of the 2D toy potential used to showcase the configuration space restriction, considerations when picking the resampling threshold, further analysis of the isopropanol SSPD runs, validations for the accuracy of the MLIP Hessians and an analysis validating the usage of the Hessian submatrix, and description and analysis of the temperature annealing experiments which showed a tighter convergence to the transition state in the CO dissociation on Co(001) (PDF)
