# Supplementary material for: Steering Langevin Dynamics toward Transition States Using Collective-Variable-Free Resampling
Source: J Chem Theory Comput. 2026 Jun 1;22(11):5797–806. doi: 10.1021/acs.jctc.6c00679 (PMC13255169; doi:10.1021/acs.jctc.6c00679)
Supplement: Supplementary file 1 [file ct6c00679_si_001.pdf]

Supplementary Information for Steering Langevin dynamics towards transition states using collective-variable-free resampling

Michael Ketter and Georg K. H. Madsen*

Institute of Materials Chemistry, TU Wien, A-1060 Vienna, Austria

E-mail: georg.madsen@tuwien.ac.at

2D toy potential

The main article rationalized the need for the configuration space restriction by using the 2D toy potential $V(x, y) = (x - 3)^4 + y^4 + 200\exp(-(10(x + 2)^2 + 10y^2)^4))$. For better interpretability of the test case, Fig. S1 encodes the number of negative eigenvalues by color.

Accuracy considerations for MLIP Hessians

Since DFT Hessians are in practice often calculated using finite difference approaches of the force, we believe that an MLIP providing an accurate fit of the forces for a universal and dense enough training database can provide accurate Hessians. To underscore this heuristically, we compared the eigenvalues of the predicted Hessians to the corresponding ground truths for all test structures in the isopropanol database used to fit the MLIPs. As it can be seen in Fig. S2, the eigenvalues show satisfactory agreement over the full range. Even if a universal and dense enough database is not available, we believe that the exploratory approach of

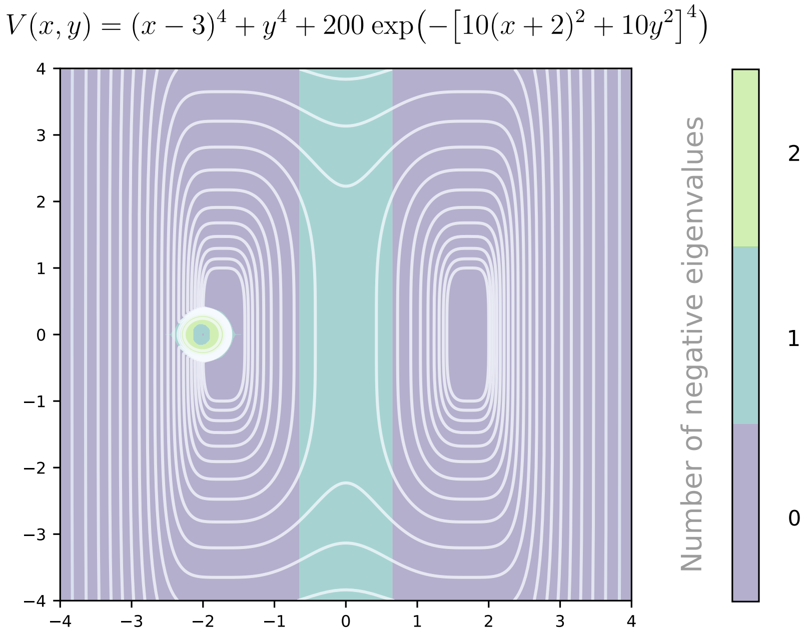

Supplementary figure S1: Encoding of the number of negative eigenvalues for the 2D toy potential on the full configuration space.

SSPD would allow a stepwise and target-oriented expansion of an initial training database by using active-learning approaches.

ESS threshold considerations

In practice, we observed two opposing influences of the ESS threshold choice on the behavior of the algorithm. For a high ESS threshold, we observed very early resampling such that the exploration of the configuration space was not sufficient enough to converge to reactive transition state regions. Because of the presence of conformational saddle points, this was especially apparent in the isopropanol test case. For low ESS thresholds, we found that the resampling was performed too infrequently to keep the ensemble in the transition state region without sliding into one of the adjacent minima. Using the LJ-7 test case we evaluated different ESS thresholds to obtain an applicable lower bound. As can be seen in Fig. S3, an ESS threshold of 0.99N was the lowest value to consistently keep the ensemble around

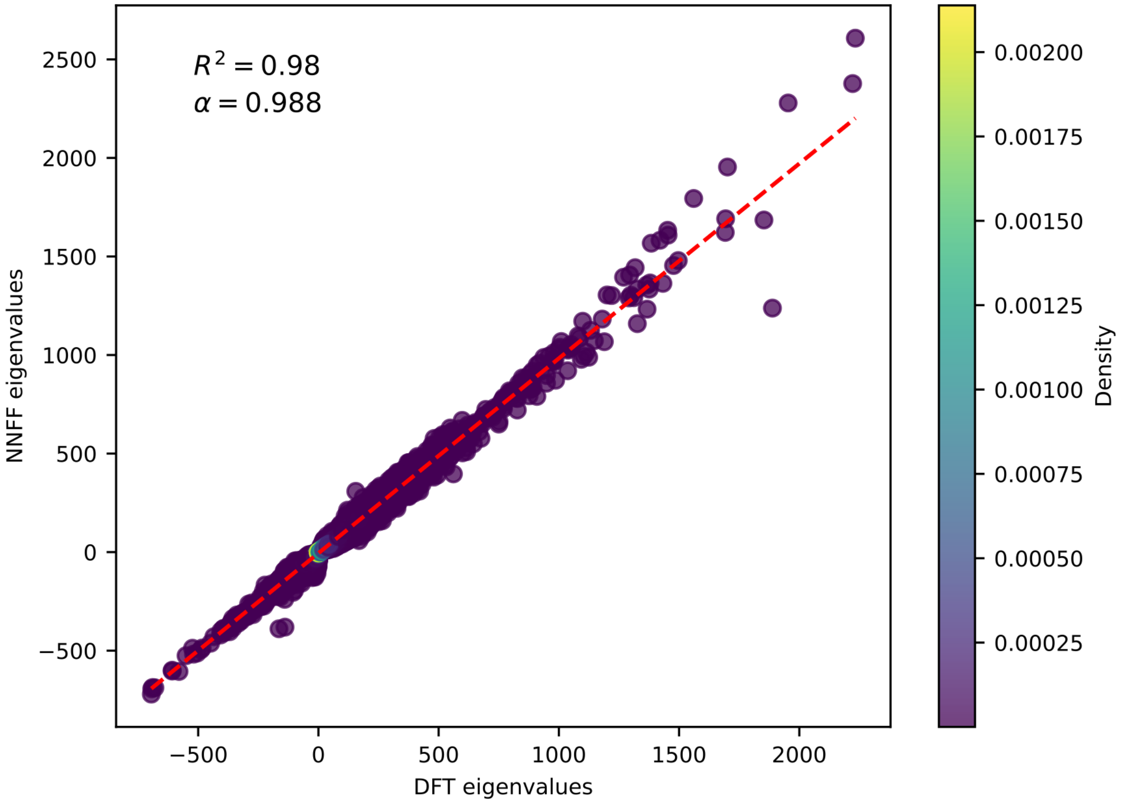

Supplementary figure S2: Parity plot comparing the eigenvalues of the DFT Hessians with the ones from the MLIP Hessian for all structures in the MLIP testset.

the transition state region. We therefore settled for an ESS threshold of 0.99N for all our experiments.

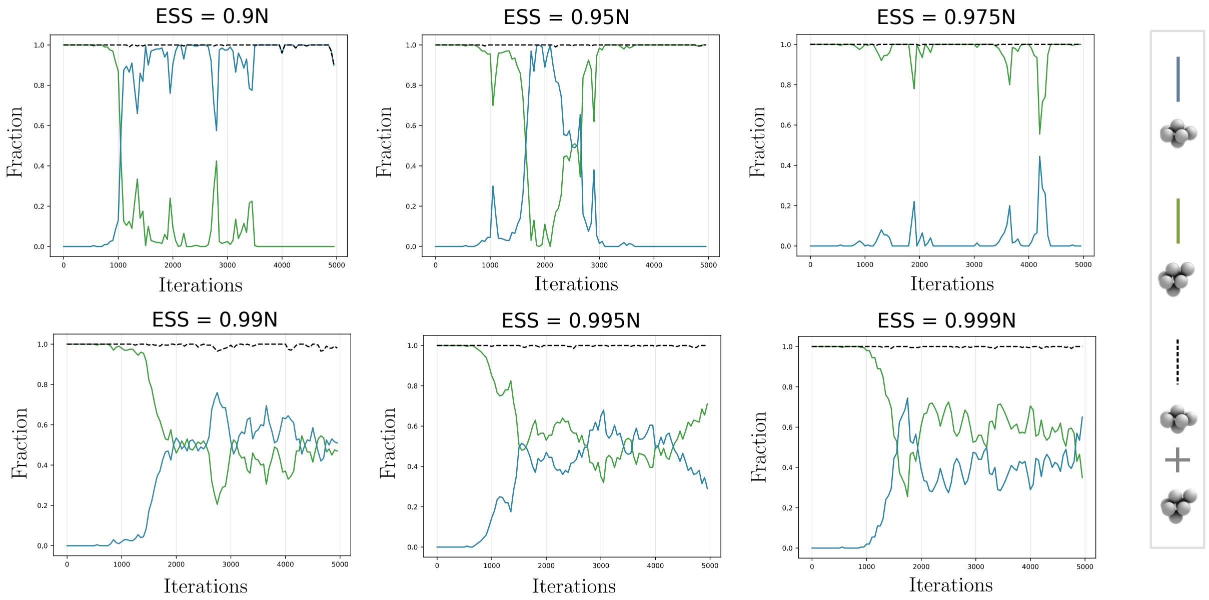

Supplementary figure S3: Analyzing the behavior of SSPD for different ESS values on the LJ-7 test system. Each run was initialized using the same random seed and with $N = 200$.

Analyzing the final Isopropanol ensembles of different runs

To show that the SSPD algorithm converges to the same TS configurations as NEB searches, we analyze the final ensemble distribution of four runs. It was visually assessed that those four runs converged to each of the reactions described in the main part of the study. For the analysis, the dimensionality reduction technique UMAP was used to embed the high-dimensional position vector into 2D space. The UMAP model was trained by applying following procedure: from each of the four relevant reactions, the NEB ground truth educt, TS and product configurations were extracted. To include the surrounding of those states, 100 perturbed configurations were generated from each extracted configuration by adding white noise. Using spherical Bessel descriptors, the resulting 1200 configurations were en-

coded in a rotationally invariant manner. The resulting descriptors were used to train the UMAP embedding.

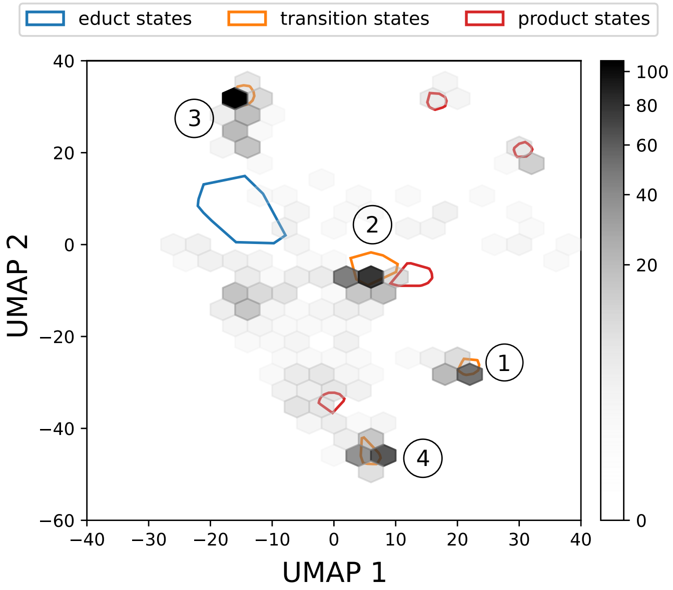

thus

Supplementary figure S4: 2D UMAP-embedding of the final ensemble of four runs converging to each of the four reactions depicted at the top.

The configurations of the final ensemble corresponding to the four SSPD runs were encoded as spherical Bessel descriptors and projected onto 2D space using the obtained UMAP embedding. To obtain an empirical estimation of the SSPD ensembles distribution, a hexbin plot was generated using the corresponding 2D data points. The hexbin-plot and the convex hulls of the UMAPs training data are depicted in Fig. S4. From this, it can be seen that the hexagons exhibiting the highest counts lie in or surround the convex hulls of the ground truth NEB TSs. Additionally, it can be seen that the hexagons corresponding to the educt- and product configurations are populated very sparsely. With that, we can conclude that SSPD successfully shifts the ensemble distribution towards the NEB TS region in all four reactions.

Verifying the usage of Hessian submatrizes

To validate the approximation to only use a submatrix of the Hessian to update the weights, Figs. S5(a) and (b) compare the ensemble distribution of the lowest eigenvalues along the trajectory for the submatrix approach and the full Hessian. Both approaches exhibit a sharp drop in the lowest eigenvalue distribution around 2000 iterations. Prior to this drop, the submatrix approach shows a constant trend of the lowest eigenvalues, while the full Hessian matrix introduces a small but steady constant decrease. This decrease can be associated with arising instabilities in the cobalt slab. Since the submatrix approach incorporates solely the positions of the CO-molecule, the instabilities of the cobalt slab are not captured.

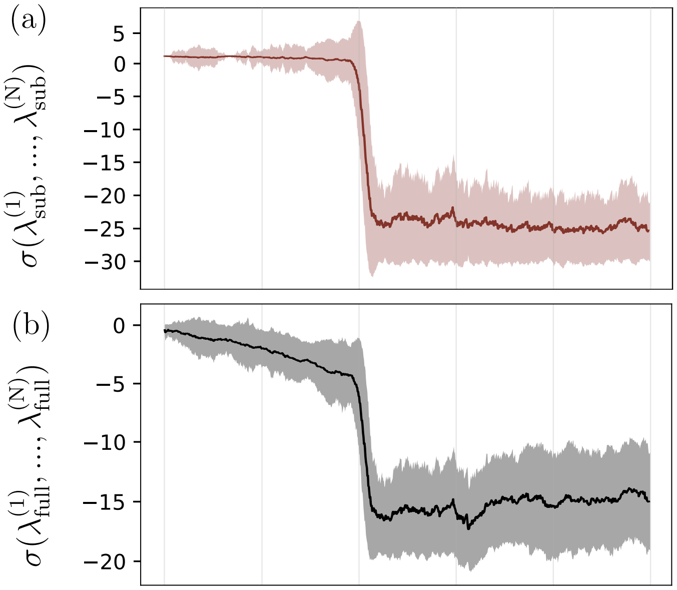

Supplementary figure S5: a, b) Time evolution of the mean and standard deviation of the lowest Hessian eigenvalues. a) analyzes the eigenvalues of the 6×6 submatrix of the Hessian, while b) analyzes the eigenvalues of the full Hessian.

CO-dissociation on Co(001)

As described, temperature annealing can be used to achieve a more concentrated convergence to the transition state regions. In practice, we found that when decreasing the temperature,

it can be necessary to increase the resampling threshold in order to prevent sliding into one of the adjacent potential energy minima. In addition, the annealing regime has to be designed such that the temperature is varied in a smooth fashion. The annealing schedule depicted in Fig. S6 follows this strategy and was tested to reduce the temperature by a factor of ten in the investigation of CO-dissociation on Co(001).

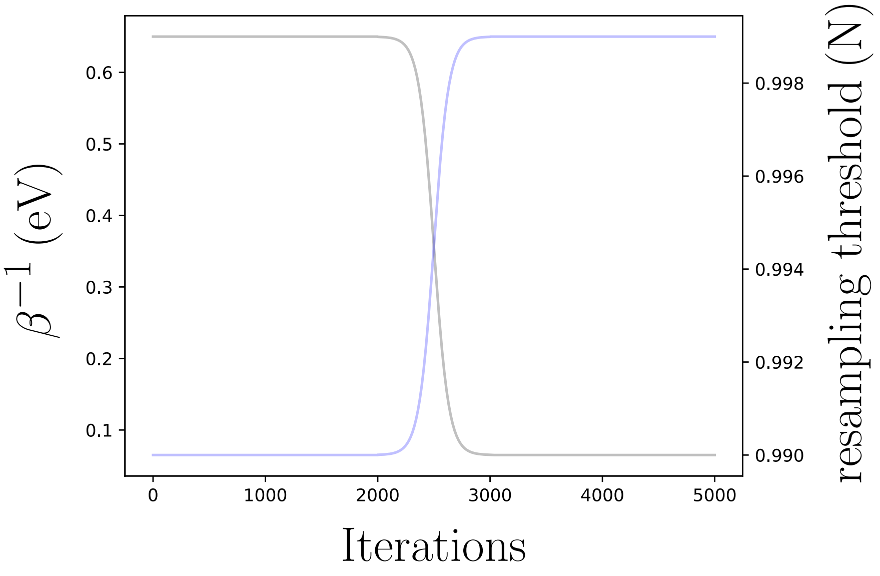

Supplementary figure S6: Temperature annealing (grey) and resampling threshold (purple) schedule used for CO-dissociation on Co(001).

By mapping the ensemble onto the introduced 2D-CV space of the reaction, Fig. S7 compares the influence of the annealing schedule to the ensemble distribution. As it can be seen, the annealing schedule concentrates the ensemble distribution much stronger on the transition state region compared to a run without the annealing schedule.

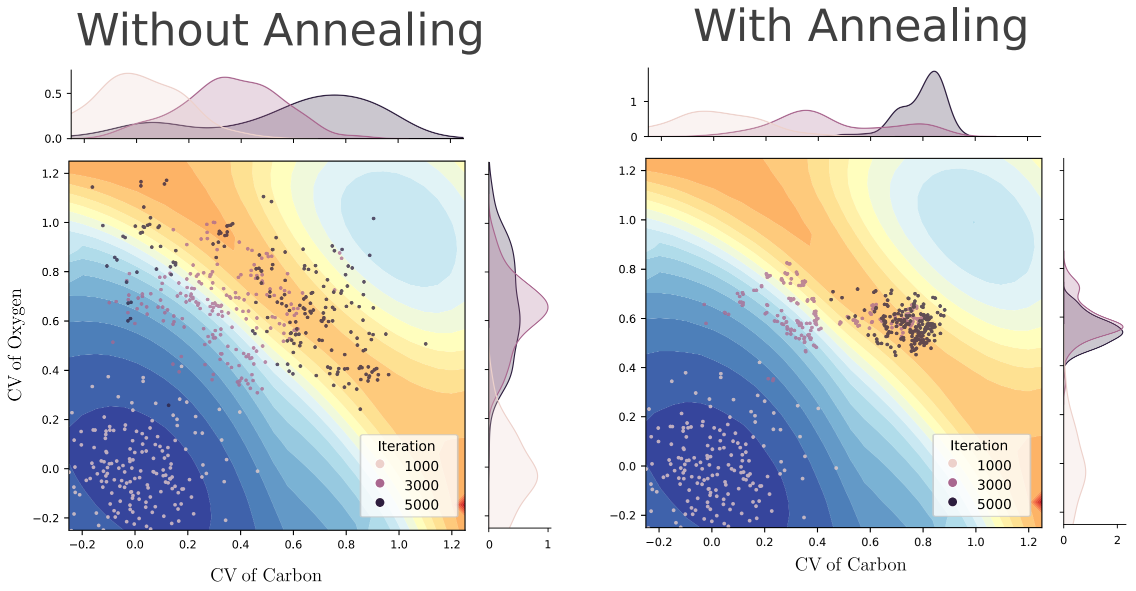

Supplementary figure S7: Comparing the resulting ensemble distribution with and without temperature annealing by mapping it onto the introduced 2D-CV space.
